# Perioperative, oncologic, and functional outcomes of robot-assisted partial nephrectomy for special types of renal tumors (hilar, endophytic, or cystic): an evidence-based analysis of comparative outcomes

**DOI:** 10.3389/fonc.2023.1178592

**Published:** 2023-04-20

**Authors:** Xiao-bin Chen, Yu-gen Li, Tao Wu, Zhong-bo Du, Chun-lin Tan, Qiang Zhang, Xiao-dong Yu

**Affiliations:** ^1^ Department of Urology, Affiliated Hospital of North Sichuan Medical College, Nanchong, China; ^2^ Department of Clinical Medicine, North Sichuan Medical College, Nanchong, China

**Keywords:** renal hilar tumors, endophytic renal tumors, cystic renal tumors, robot-assisted partial nephrectomy, meta-analysis

## Abstract

**Purpose:**

This study aims to perform a pooled analysis to compare the outcomes of robot-assisted partial nephrectomy (RAPN) between complex tumors (hilar, endophytic, or cystic) and non-complex tumors (nonhilar, exophytic, or solid) and evaluate the effects of renal tumor complexity on outcomes in patients undergoing RAPN.

**Methods:**

Four databases were systematically searched, including Science, PubMed, Web of Science, and Cochrane Library, to identify relevant studies published in English up to December 2022. Review Manager 5.4 was used for statistical analyses and calculations. The study was registered with PROSPERO (Registration number: CRD42023394792).

**Results:**

In total, 14 comparative trials, including 3758 patients were enrolled. Compared to non-complex tumors, complex tumors were associated with a significantly longer warm ischemia time (WMD 3.67 min, 95% CI 1.78, 5.57; p = 0.0001), more blood loss (WMD 22.84 mL, 95% CI 2.31, 43.37; p = 0.03), and a higher rate of major complications (OR 2.35, 95% CI 1.50, 3.67; p = 0.0002). However, no statistically significant differences were found between the two groups in operative time, length of stay, transfusion rates, conversion to open nephrectomy and radical nephrectomy rates, estimated glomerular filtration rate (eGFR) decline, intraoperative complication, overall complication, positive surgical margins (PSM), local recurrence, and trifecta achievement.

**Conclusions:**

RAPN can be a safe and effective procedure for complex tumors (hilar, endophytic, or cystic) and provides comparable functional and oncologic outcomes to non-complex tumors.

**Systematic review registration:**

https://www.crd.york.ac.uk/prospero/display_record.php?RecordID=394792, identifier CRD42023394792.

## 1 Introduction

Partial nephrectomy (PN) is currently considered the optimal treatment for small renal tumors, as recommended by the AUA and EAU guidelines (1, 2). In addition to achieving comparable surgical outcomes and cancer control to radical nephrectomy, PN allows for the preservation of the nephrons (3). The field of PN has seen innovative advances with the introduction of robotic technology, and open PN has gradually given way to robot-assisted partial nephrectomy (RAPN) (4). Furthermore, Choi et al. (5) have demonstrated that RAPN was associated with a shorter warm ischemic time, lower rate of conversion to open nephrectomy, and less estimated glomerular filtration rate (eGFR) decline compared to laparoscopic PN. Recently, RAPN has increasingly been applied to technically challenging complex tumors.

The renal tumor complexity mainly depends on some tumor-associated factors, such as tumor size and type (endophytic, hilar, or cystic) (6). In renal hilar tumors, the mass is in close proximity to the urinary collecting system and the major renal vessels, which adds to the difficulty of the PN (7). Due to the surgeon being unable to identify the tumor location and size, PN for endophytic renal tumors is very challenging (8). In addition, performing PN for cystic renal tumors is also more difficult than for solid renal tumors because of the risk of cyst wall damage and tumor cell spillage (9). Garisto et al. (10) reported that RAPN provided acceptable results in terms of perioperative, functional and oncological outcomes for complex tumors (RENAL score > 9). The RENAL score (11) is a scoring tool to predict the difficulty of nephrectomy, but it has some shortcomings. It does not sufficiently evaluate some factors closely related to the complexity of renal tumors, such as renal hilar tumors and cystic renal tumors. Therefore, the effectiveness and safety of RAPN for these types of tumors remain controversial.

This systematic review and meta-analysis aim to integrate the data from comparative studies to evaluate the efficacy and safety of RAPN for complex tumors (hilar, endophytic, or cystic), providing guidance for clinical decision-making.

## 2 Methods

The present study was performed in accordance with the Preferred Reporting Items for Systematic Reviews and Meta-Analyses (PRISMA) statement 2020 (12, 13), and was registered in PROSPERO (Registration number: CRD42023394792).

### 2.1 Literature search strategy, study selection and data collection

Four databases, including Science, PubMed, Web of Science, and Cochrane Library, were systematically searched to identify fully published studies till December 2022. The search terms were as follows: ((Renal hilar tumors OR hilar tumors OR renal hilar masses) AND (Endophytic renal tumors OR endophytic renal masses) AND (Cystic renal cell carcinoma OR cystic renal tumors OR cystic renal masses) AND (Robotic partial nephrectomy OR Robot-assisted partial nephrectomy OR Robot-assisted nephron-sparing surgery)). Furthermore, the relevant references were manually searched to avoid omissions and expand the search scope.

The PICOS approach was used to define the inclusion criteria (1): all the patients were diagnosed with localized renal tumors; (2): in the experimental group, the patients were diagnosed with renal hilar tumors, endophytic renal tumors, or cystic renal tumors, and underwent RAPN; (3): in the control group, patients were diagnosed with renal nonhilar tumors, exophytic renal tumors, or solid renal tumors, and underwent RAPN; (4): the studies measured the perioperative, complication, renal functional and oncologic outcomes; (5): randomized controlled trials (RCTs), prospective or retrospective comparative studies. The exclusion criteria included: (1) non-comparative studies and duplicate studies, (2) letters, comments, case reports and unpublished studies, and (3) studies that did not contain the required data for meta‐analysis.

Two reviewers independently extracted the data from each qualified literature. The following data were extracted: (1) first author, year of publication, center, and country. (2) Age, body mass index (BMI), sample size, preoperative eGFR, PADUA score, and follow-up period. (3) Perioperative outcomes, including operative time, blood loss, transfusion rates, hospital stay, warm ischemia time, conversion to open nephrectomy and radical nephrectomy rates, intraoperative complications, major complications (defined as Clavien grade ≥ 3), and overall complications (defined as Clavien grade ≥ 1) (14) (4). Renal functional and oncologic outcomes, including eGFR decline, positive surgical margins (PSM), local recurrence, trifecta achievement: margin status (negative), warm ischemia time (< 25 min), and complications (Clavien grade ≤ 2), tumor size, tumor stage and pathology. Any discrepancies and disagreements were resolved by reaching a consensus with a third reviewer.

In these studies, the risk of bias in non-randomized studies of interventions (ROBINS-I) was used to assess the quality of the literature (15). Two independent reviewers estimated the quality of the included studies, and any discrepancies were solved through discussion.

### 2.2 Statistical analysis

The statistical analysis of this study was processed using Cochrane Collaborative RevMan5.4 software. The weighted mean difference (WMD) was calculated for continuous variables, whereas the odds ratio (OR) was calculated for dichotomous variables, and the results were presented with 95% confidence intervals (CI). Furthermore, the I^2^ test was used to measure the heterogeneity of each indicator among the studies (16), and statistical significance was defined as p < 0.05. For outcomes with significant heterogeneity (I^2^ > 75%), sensitivity analysis was performed by excluding one study from the pooled effect at a time to identify the source of heterogeneity and to assess the robustness.

### 2.3 Subgroup analysis

A subgroup analysis was performed to compare the different tumor types: renal hilar vs. renal nonhilar tumors, endophytic vs. exophytic renal tumors, and cystic vs. solid renal tumors.

### 2.4 Publication bias

Publication bias was examined using the Begg’s method funnel plot.

## 3 Results

### 3.1 Baseline characteristics

**Figure 1:**
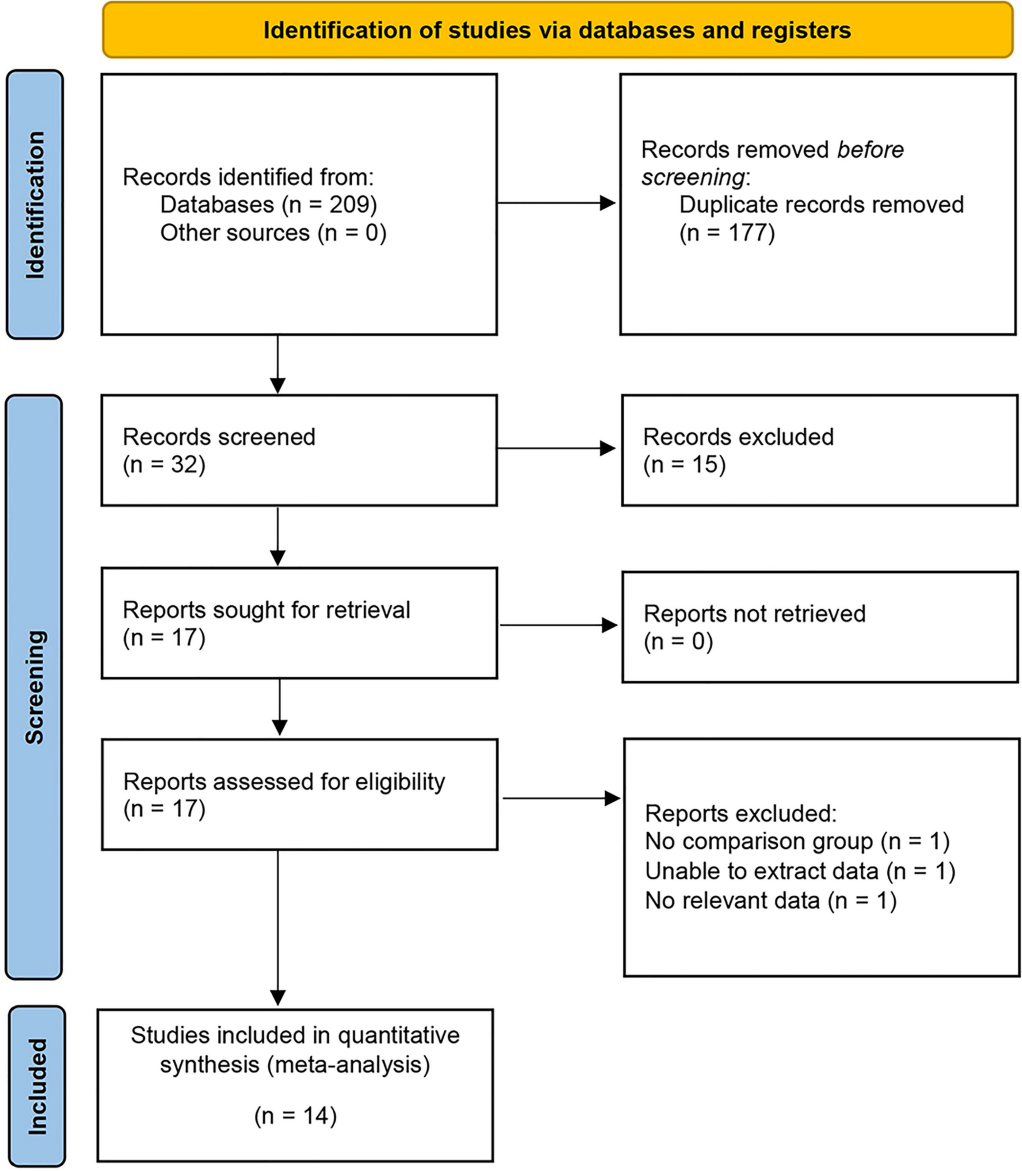
PRISMA flow diagram for the systematic review.

Initially, a comprehensive electronic search yielded 209 studies, which were subsequently reduced to 32 after the removal of duplicate entries. A preliminary evaluation of the titles and abstracts of these studies led to the selection of 14 studies, which involved a total of 3758 patients, for inclusion in our meta-analysis (**Figure 1**) (17–30). All 14 non-RCTs were retrospective comparisons, with three studies being multi-institutional (21, 23, 29), while the others were single-center. The studies were conducted in different countries, including the USA, Korea, China, and Japan, with a follow-up period of between 3.3 to 48 months. The key characteristics of all patients included in each study are summarized in **Table 1** and **Table 2** (including sample size, age, BMI, gender, tumor diameter, tumor site, preoperative eGFR, RENAL score and tumor types). **Table 3** displays the oncologic outcomes.

**Table 1:** The trials included in the systemic review.

Reference	Year	Country	Center	Patients		Age(y)		Male/Female		BMI (kg/m^2^)	
				Complex group	Non-complex group	Complex group	Non-complex group	Complex group	Non-complex group	Complex group	Non-complex group
Tyagi	2021	India	single-center	41	41	50.4(12.7)	51.5(11.7)	24/17	25/16	24.9(3.3)	24.4(3.0)
Liu	2021	China	single-center	75	98	55(16)	55.5(13.25)	43/32	71/27	NA	
Lu	2018	China	single-center	30	170	52.4(15.3)	58(13.5)	14/16	99/71	23.7(3.3)	25.4(3.9)
Eyraud	2013	USA	single-center	70	294	58(14.07)	59(11.11)	45/25	170/124	29.39(5.19)	29.29(6.01)
Dulabon	2011	USA	multi-institutional	41	405	59.3(12.8)	60.0(11.3)	29/12	233/172	28.6(6.3)	30.2(6.4)
Motoyama	2022	Japan	single-center	26	127	64.5(12.5)	68(15.75)	18/8	83/44	24.8(5.43)	24.2(8.05)
Carbonara	2020	USA and Europe	multi-institutional	147	510	57.7(11.8)	60.9(12.7)	93/54	296/214	27.4(5.7)	27.7(5.2)
Komninos	2014	Korea	single-center	45	64	50(9.63)	51(10.37)	31/14	30/34	26.1(3.33)	25.5(3.7)
Autorino	2014	USA	single-center	65	179	56(1.4)	61.2(0.9)	31/34	111/68	29.4(6.3)	31.2(7.4)
Yagisawa	2022	Japan	single-center	83	83	55(14)	54(13)	65/18	60/23	24(4)	23(4)
Zennami	2021	Japan	single-center	46	271	58(14.07)	62(12.59)	33/13	202/69	23.5(2.67)	23.8(3.04)
Raheem	2016	Korea	single-center	32	263	51.3(11.3)	52.1(12.5)	16/16	168/95	24.4(2.6)	24.7(3.5)
Novara	2016	Europe	multi-institutional	54	411	62(12.22)	58(12.59)	36/18	281/130	NA	
Akca	2014	USA	single-center	55	55	55.82(14.9)	58.67(12.1)	26/29	28/27	29.11(54)	30.22(5.6)

BMI, Body mass index; Mean (SD).

**Table 2:** The trials included in the systemic review.

Reference	Tumor site (Lt/Rt)		Tumor diameter (cm)		Preoperative eGFR (ml/min/1.73 m)		RENAL score		Type	Follow-up duration (month)	
	Complex group	Non-complex group	Complex group	Non-complex group	Complex group	Non-complex group	Complex group	Non-complex group		Complex group	Non-complex group
Tyagi	22/19	18/23	4.4(1.6)	3.5(1.5)	100.8(41.8)	92.3(28.0)	7.9(1.7)	7.8(1.7)	Renal hilar tumors	Range: 3-12	
Liu	NA		4.8(1.98)	4.5(1.65)	113.9(33.08)	121.5(11.6)	9(1.5)	6(1.75)	Renal hilar tumors	Mean: 30	
Lu	14/16	74/96	4.8(2)	3.7(1.8)	94.1(13.3)	86.5(25.3)	9(1.2)	7.4(1.7)	Renal hilar tumors	Mean: 28 (range: 3-12)	Mean: 32.3 (range: 3-12)
Eyraud	NA		3.9(1.63)	2.6(1.26)	84.8(19.26)	85.6(21.04)	Low: 0; Intermediate: 29; High: 41	Low: 146; Intermediate: 132; High: 16	Renal hilar tumors	Mean: 7.4 (range: 1.3-18.5)	
Dulabon	NA		3.46(1.35)	2.88(1.53)	NA		NA		Renal hilar tumors	Range: 3-45	
Motoyama	8/18	63/64	1.9(1.0)	2.9(1.75)	NA		9(1.25)	6(1.5)	Endophytic renal tumors	NA	
Carbonara	NA		4.2(2.5)	3.2(4.1)	84.2(22.7)	83.6(21.4)	10(1.48)	4(1.48)	Endophytic renal tumors	21.6(20)	32.3(25.4)
Komninos	26/19	29/35	2.6(1.56)	2.5(2.96)	84.4(10.37)	90(14.07)	9(1.48)	5.5(2.22)	Endophytic renal tumors	Mean: 48 (range: 20-59)	Mean: 38 (range: 16-63)
Autorino	32/33	90/89	2.6(1.0)	3.7(2.1)	89.6(22.9)	80.1(23.2)	8.7(1.4)	6.4(2.2)	Endophytic renal tumors	12.6(11.0)	14.5(13.8)
Yagisawa	NA		2.8(1.3)	3.0(1.3)	67(17)	69(17)	Low: 17; Intermediate: 59; High: 8	Low: 32; Intermediate: 36; High: 15	Cystic renal tumors	23(22)	21(18)
Zennami	NA		3.2(1.48)	2.9(1.11)	68.6(16.67)	69.5(15.41)	Low: 19; Intermediate: 23; High: 4	Low: 120; Intermediate: 137; High: 14	Cystic renal tumors	Mean: 41	Mean: 37
Raheem	NA		3.7(1.9)	3.3(1.8)	88(14.82)	90(8.89)	Low: 5; Intermediate: 11; High: 16	Low: 67; Intermediate: 91; High: 104	Cystic renal tumors	Mean: 58 (range: 24–63)	Mean: 46 (range: 24–60)
Novara	NA		3.45(2.16)	3.2(1.30)	79(20.74)	81(19.26)	Low: 13; Intermediate: 28; High: 13	Low: 201; Intermediate: 118; High: 92	Cystic renal tumors	Mean: 10 (IQR: 3–24)	Mean: 6 (IQR: 3–19)
Akca	25/30	29/26	2.7(2.6)	3.1(2.0)	89.6(38.1)	80.1(27.1)	9(2.96)	9(2.96)	Cystic renal tumors	Mean: 10(IQR: 22)	Mean: 7(IQR: 9)

eGFR, estimated glomerular filtration rate; Mean (SD); IQR, interquartile range.

**Table 3:** Oncologic outcomes.

Reference	Tumor stage		Tumor pathology	
	Complex tumor group	Non-complex tumor group	Complex tumor group	Non-complex tumor group
Tyagi	pT1a:12; pT1b:21; pT2a:1; pT2b:2; pT3a:1	pT1a:22; pT1b:14; pT2a:2; pT2b:2; pT3a:0	Benign: 3; Malignant: 38	Benign: 4; Malignant: 37
Liu	pTa:24; pT1b:39; pT2a:8	pTa:59; pT1b:31; pT2a:6	Clear cell: 64; Papillary: 3; Chromophobe: 4; Others: 4	Clear cell: 90; Papillary: 1; Chromophobe: 6; Others: 1
Lu	pTa:9; pT1b:5; pT2:0; pT3a:2	pTa:93; pT1b:20; pT2:1; pT3a:10	Clear cell: 16;lymphovascular invasion:2	Clear cell: 117;lymphovascular invasion:5
Eyraud	pT1a:26; pT1b:19; pT2:1; pT3a:7	pT1a:160; pT1b:30; pT2:4; pT3a:11	Clear cell: 53	Clear cell: 205
Dulabon	pT1a:23; pT1b:6; pT2:1; pT3a:5; pT3b:2	pT1a:248; pT1b:36; pT2:3; pT3a:8; pT3b:1	Clear cell: 29; Papillary: 6; Chromophobe: 1; Others: 0	Clear cell: 193; Papillary: 67; Chromophobe: 26; Others: 5
Motoyama	NA		Clear cell: 18; Others: 2; Benign: 6	Clear cell: 75; Others: 24; Benign: 28
Carbonara	pT1a:68; pT1b:33; pT2a:13; pT2b:2; pT3a:10	pT1a:307; pT1b:70; pT2a:9; pT2b:4; pT3a:18	Benign: 31; Malignant: 116	Benign: 121; Malignant: 389
Komninos	pT1a:30; pT1b:9; pT2:1; pT3a:0	pT1a:30; pT1b:10; pT2:4; pT3a:2	Benign: 5; Malignant: 40	Benign: 18; Malignant: 46
Autorino	pT1a:47; pT1b:3; pT2:0; pT3a:2	pT1a:84; pT1b:41; pT2:4; pT3a:11	Benign: 17; Malignant: 48	Benign: 40; Malignant: 139
Yagisawa	NA		NA	
Zennami	pT1a:34; pT1b:3; pT2:0; pT3a:0	pT1a:226; pT1b:20; pT2:1; pT3a:5	Clear cell: 33; Papillary: 2; Chromophobe: 1; Others: 1; Benign: 9	Clear cell: 205; Papillary: 24; Chromophobe: 18; Others: 5; Benign: 19
Raheem	cT1a:18; cT1b:13; cT2a:1; cT2b:0	cT1a:195; cT1b:58; cT2:8; cT3a:2	Benign: 3; Malignant: 29	Benign: 46; Malignant: 217
Novara	pT1a:33; pT1b:6; pT2:0; pT3a:4	pT1a:241; pT1b:54; pT2:3; pT3a:23	Clear cell: 31; Papillary: 10; Chromophobe: 1; Others: 1; Benign: 11	Clear cell: 251; Papillary: 46; Chromophobe: 25; Others: 5; Benign: 84
Akca	pT1a:14; pT1b:12; pT2a:1; pT3a:3	pT1a:28; pT1b:12; pT2a:0; pT3a:7	Clear cell: 18; Papillary: 6; Chromophobe: 2; Others: 4;	Clear cell: 21; Papillary: 16; Chromophobe: 7; Others: 3;

No significant difference was found in age (p = 0.06), tumor diameter (p = 0.26), BMI (p = 0.14) and preoperative eGFR (p = 0.86). However, the tumor diameter was significantly larger in the renal hilar tumor subgroup compared to the renal nonhilar tumors subgroup (p < 0.0001) (**Table S1**).

### 3.2 Assessment of quality

A comparative analysis was performed on all the included studies, revealing a moderate risk of bias (**Table S2**).

### 3.3 Outcome analysis

#### 3.3.1 Perioperative effectiveness

**Figure 2:**
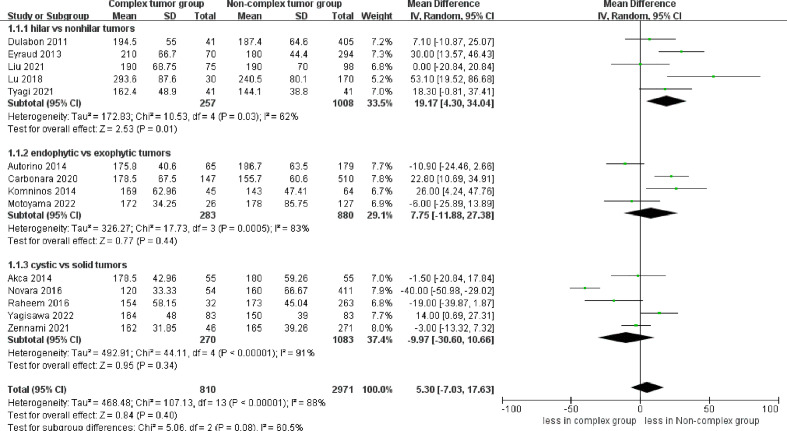
Forest plots of perioperative outcome- operative time.

**Figure 3:**
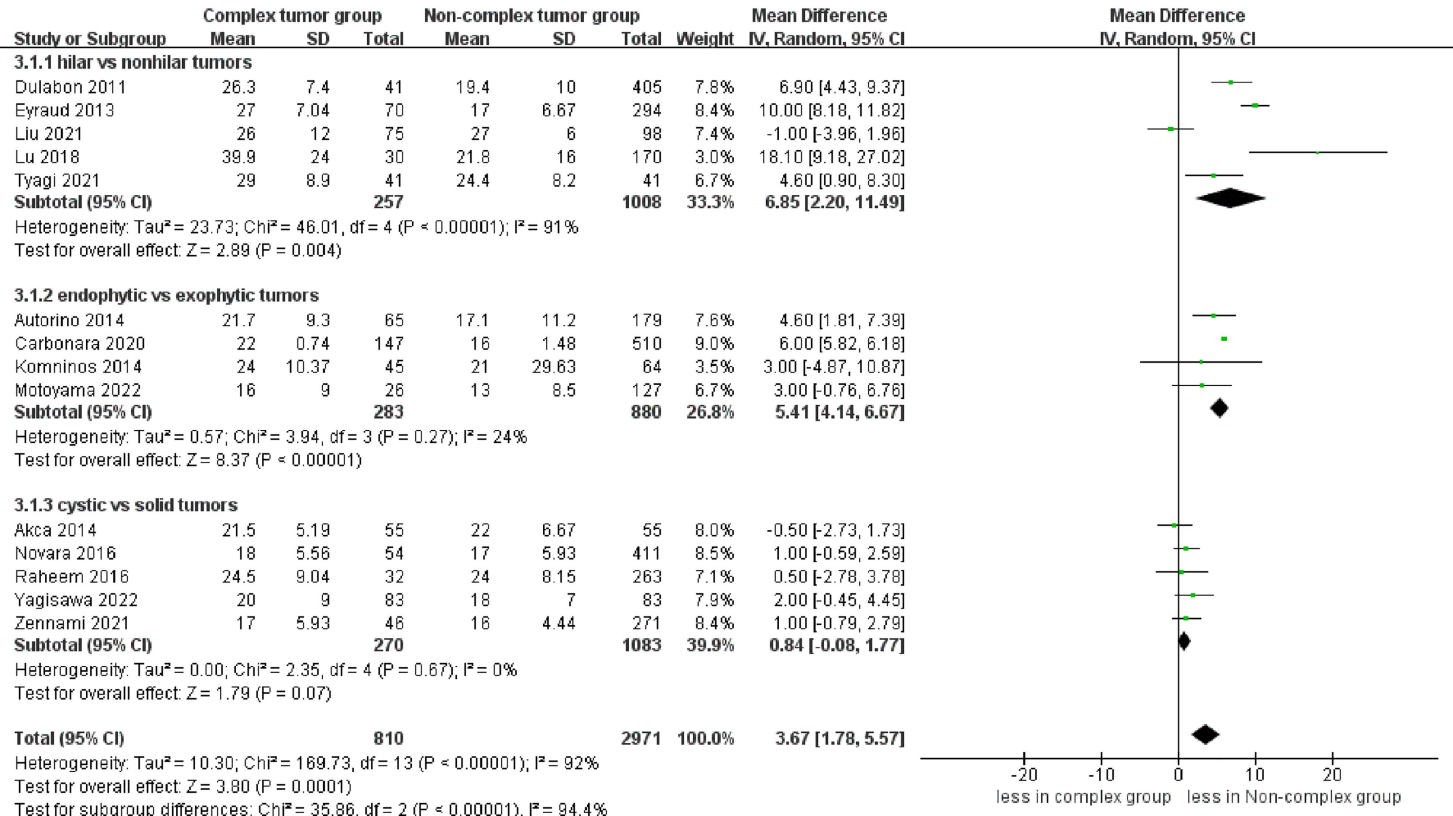
Forest plots of perioperative outcome- warm ischemia time.

The pooled results demonstrated no difference in operative time (14 studies; p = 0.40) between the complex and non-complex tumor groups (17–30). However, the subgroup analysis indicated that renal hilar tumors had a longer operative time than renal nonhilar tumors (WMD 19.17 min, 95% CI 4.30, 34.04; p = 0.01) (**Figure 2**). The meta‐analysis included 14 studies that reported the warm ischemia time (17–30). The combined results revealed that the complex tumor group was associated with a longer warm ischemia time than the non-complex tumor group (WMD 3.67 min, 95% CI 1.78, 5.57; p = 0.0001). Similar results were found in renal hilar and endophytic renal tumors groups (renal hilar tumors: WMD 6.85 min, 95% CI 2.20, 11.49; p = 0.004; endophytic renal tumors: WMD 5.41 min, 95% CI 4.14, 6.67; p < 0.00001) (**Figure 3**). The cumulative analysis revealed no significant difference in length of hospital stay between the two groups (14 studies; p = 0.69) (17–30). Furthermore, the subgroup analysis demonstrated no statistically significant differences in the length of hospital stay among the three subgroups (**Figure S1**).

**Figure 4:**
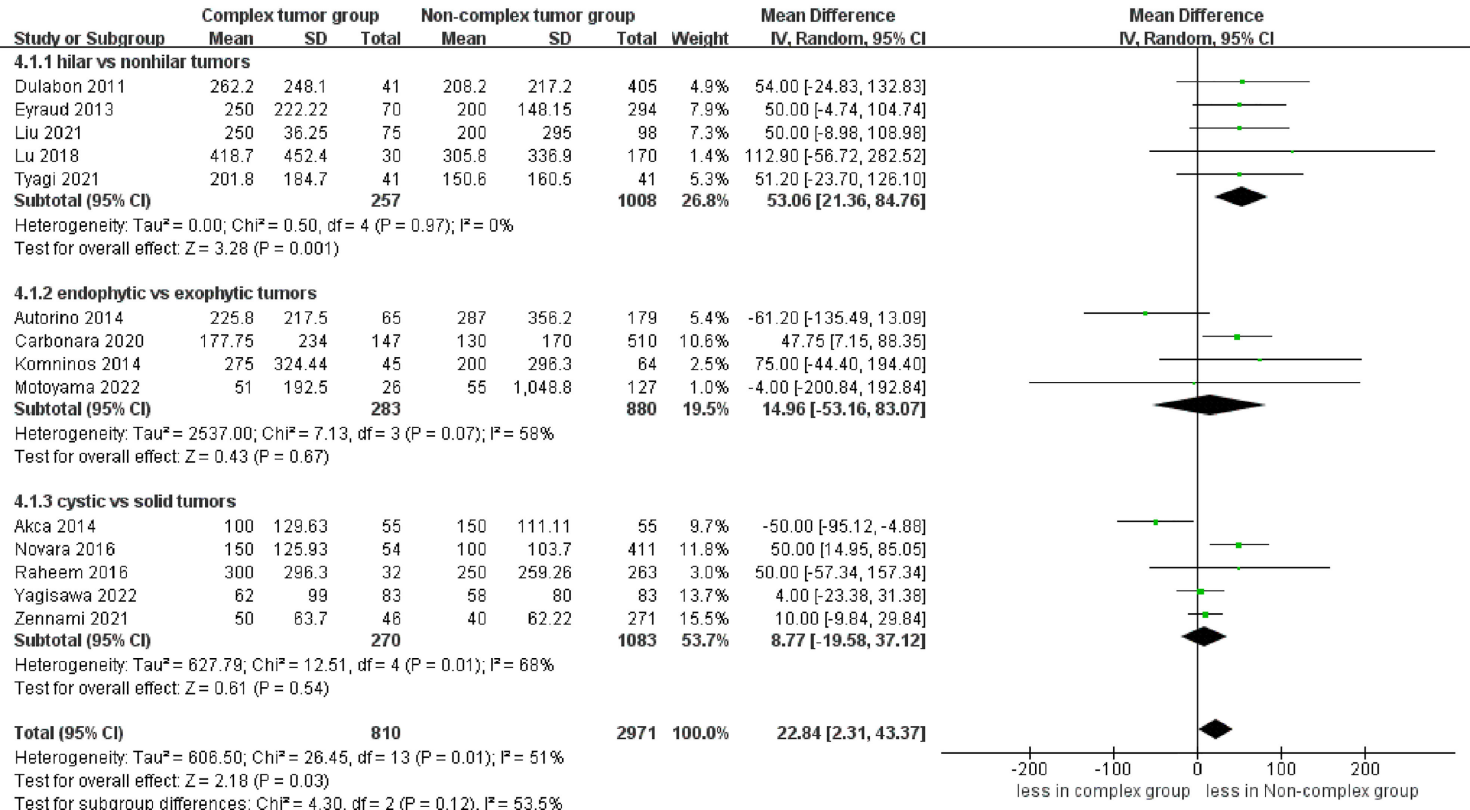
Forest plots of perioperative outcome-blood loss.

However, significantly more blood loss was observed in the complex tumor group compared with the non-complex tumor group (14 studies; WMD 22.84 mL, 95% CI 2.31, 43.37; p = 0.03) (17–30). The subgroup analysis demonstrated no statistically significant differences in blood loss between the endophytic renal tumors and the cystic renal tumors compared to the non-complex group (p = 0.67; p = 0.54) (**Figure 4**). Transfusion rates were reported in 12 studies (17–26, 28, 30). No statistically significant difference in transfusion rates was observed between the two groups (p = 0.05) (**Figure S2**). Similarly, the cumulative analysis revealed no significant difference in the prevalence of conversion to open nephrectomy and radical nephrectomy rates between the two groups (five studies; p = 0.23 and six studies; p = 0.23) (**Figure S3**) (19–22, 30) (19–22, 27, 28);.

#### 3.3.2 Complications

**Figure 5:**
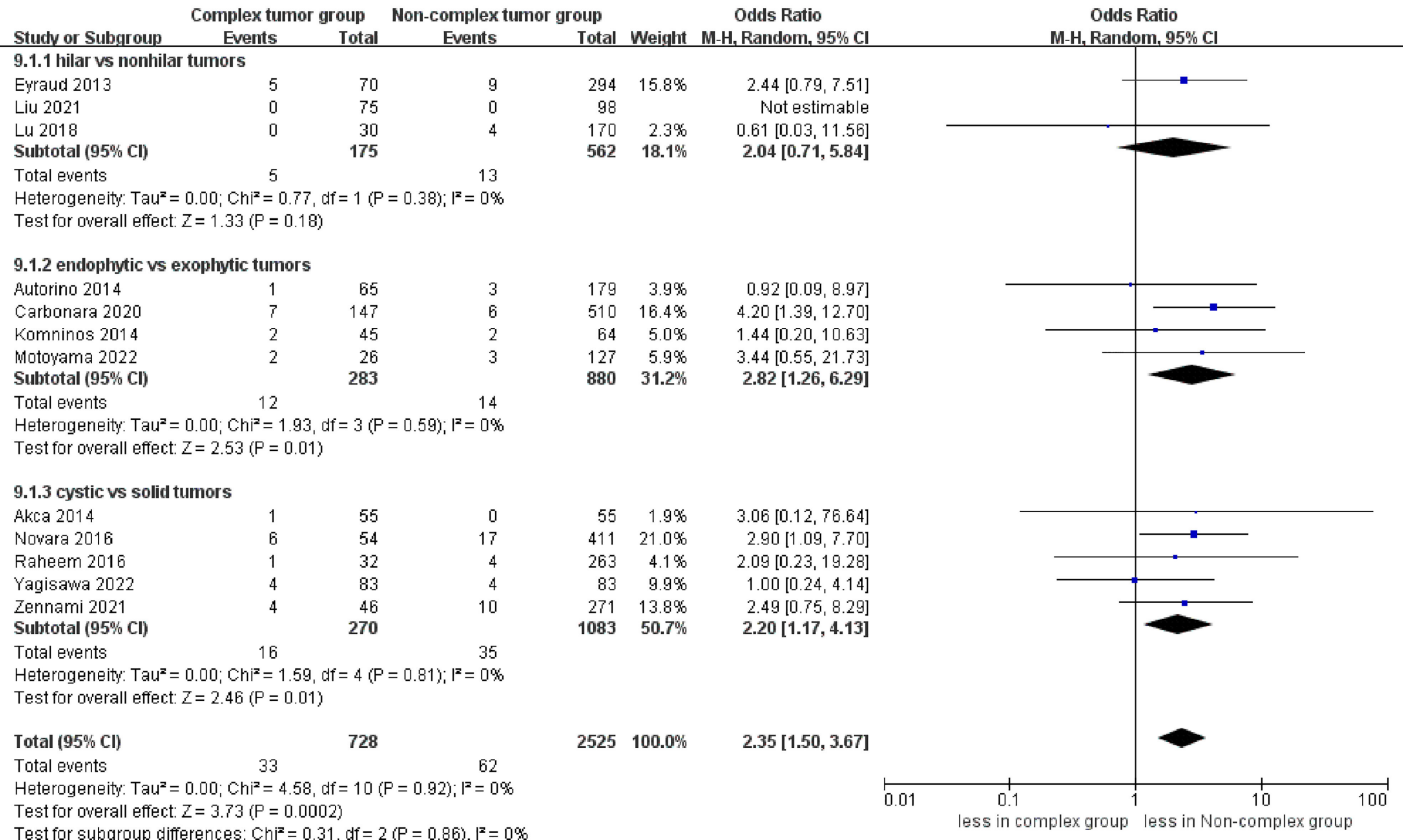
Forest plots of complication- major complications.

**Figure 6:**
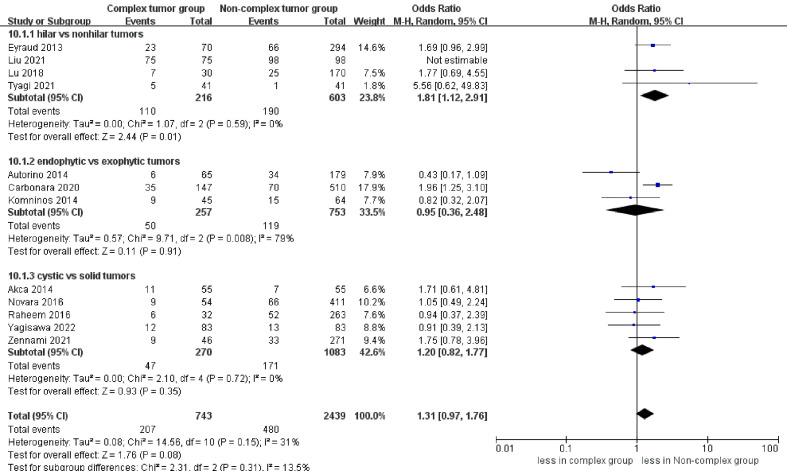
Forest plots of complication- overall complications.

No statistically significant difference in intraoperative complications was observed between the two groups (seven studies; p = 0.49) (**Figure S4**) (23–25, 27–30). However, the complex group had more major complications compared to the non-complex group (12 studies; OR 2.35, 95% CI 1.50 to 3.67, p = 0.0002) (18–20, 22–30), while the subgroup analysis revealed that no significant difference in major complications between renal hilar and nonhilar tumors (p = 0.18) (**Figure 5**). Overall complications occurred in 27.9% (207 of 743 cases) of patients in the complex group and 20.0% (480 of 2439 cases) of patients in the non-complex group. The cumulative analysis revealed no significant difference in overall complications between the two groups (13 studies; p = 0.08) (**Figure 6**) (17–20, 22–30).

#### 3.3.3 Renal functional and oncologic outcomes

**Figure 7:**
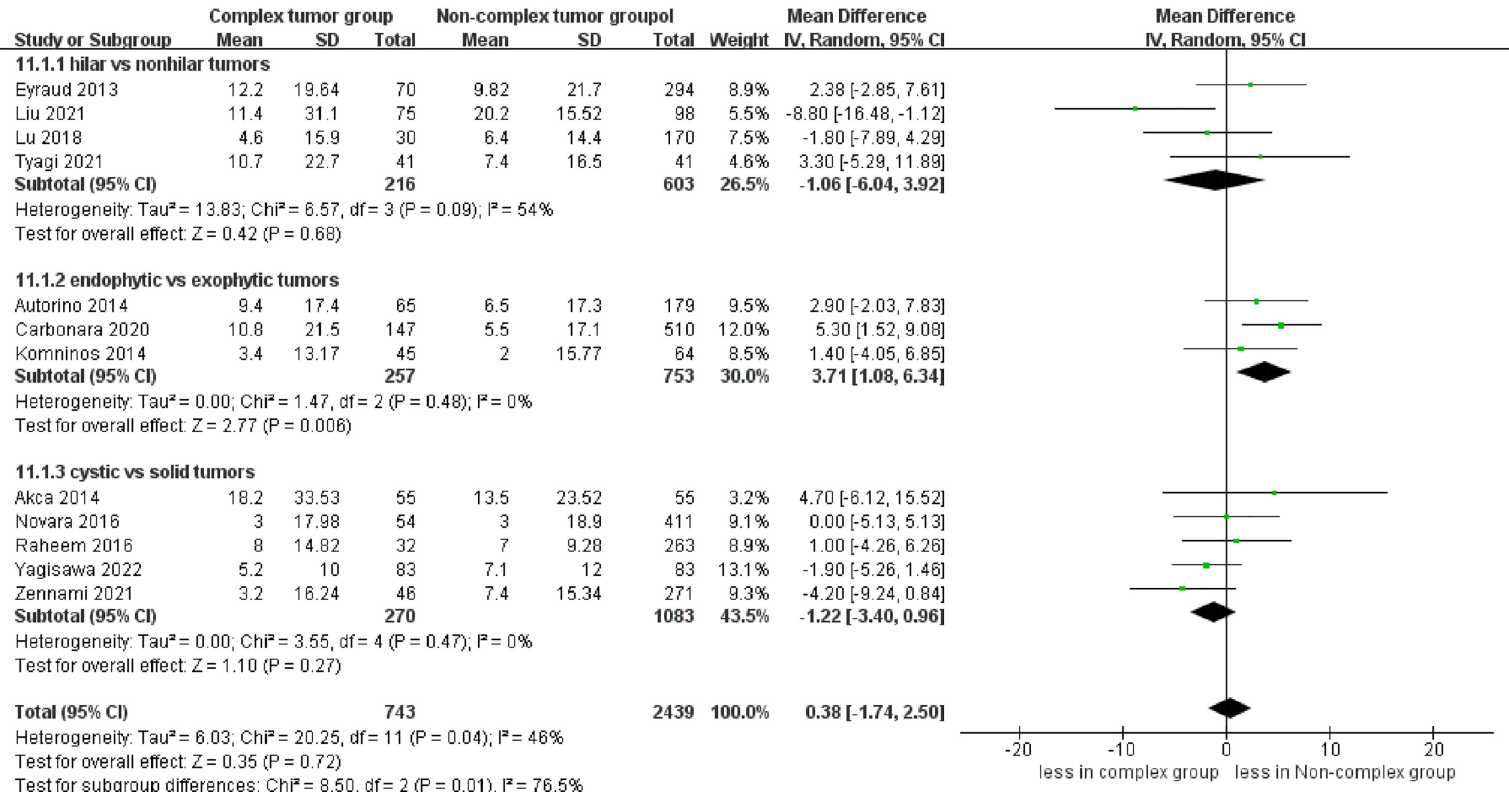
Forest plots of renal functional outcome- eGFR decline.

eGFR decline was reported in 12 studies (17–20, 23–30), demonstrating no significant differences between the two groups (p = 0.72). However, the subgroup analysis revealed that endophytic renal tumors were associated with a larger eGFR decline compared to exophytic renal tumors (WMD 3.71 mL/min/1.73 m, 95% CI 1.08, 6.34; p = 0.006) (**Figure 7**).

**Figure 8:**
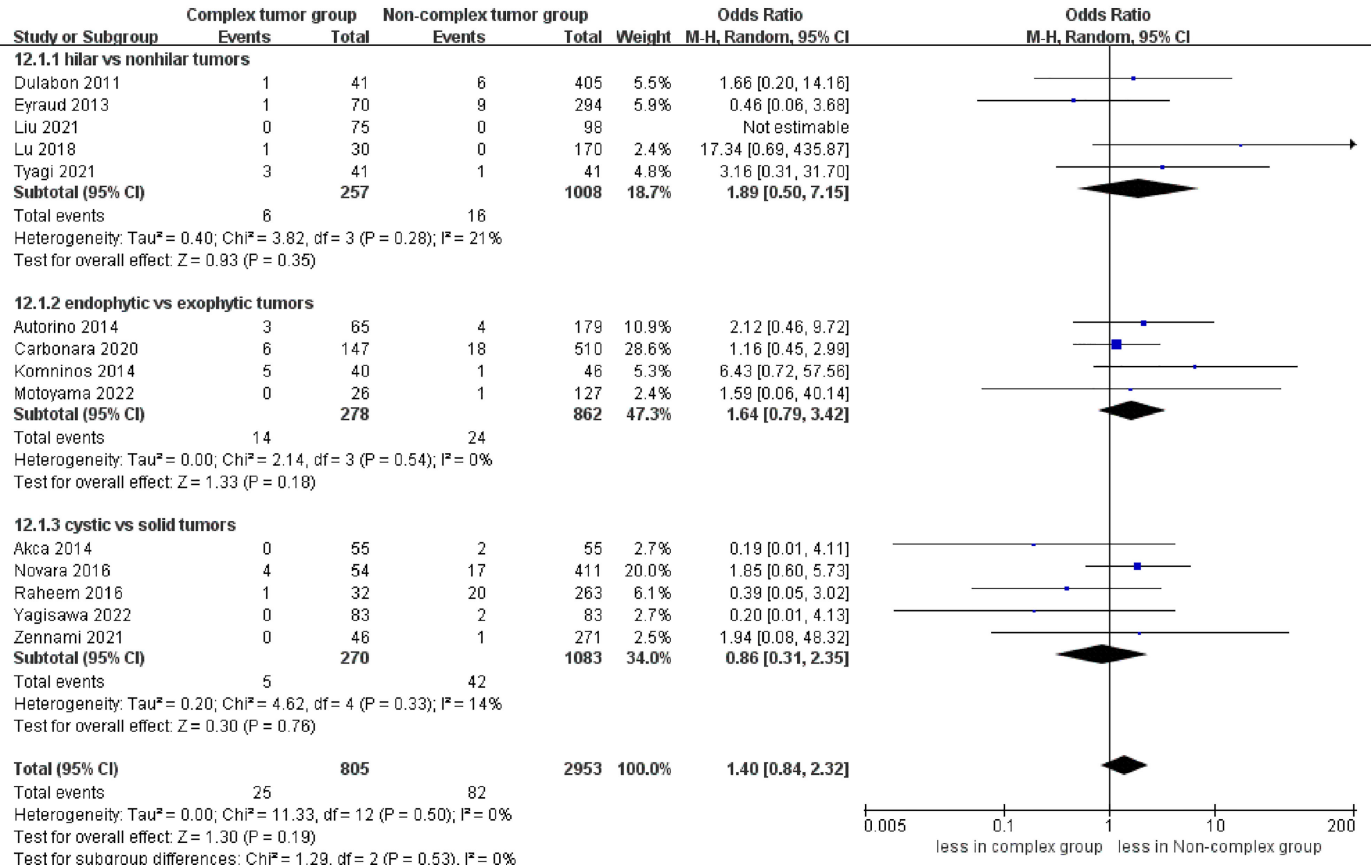
Forest plots of oncologic outcome-PSM.

**Figure 9:**
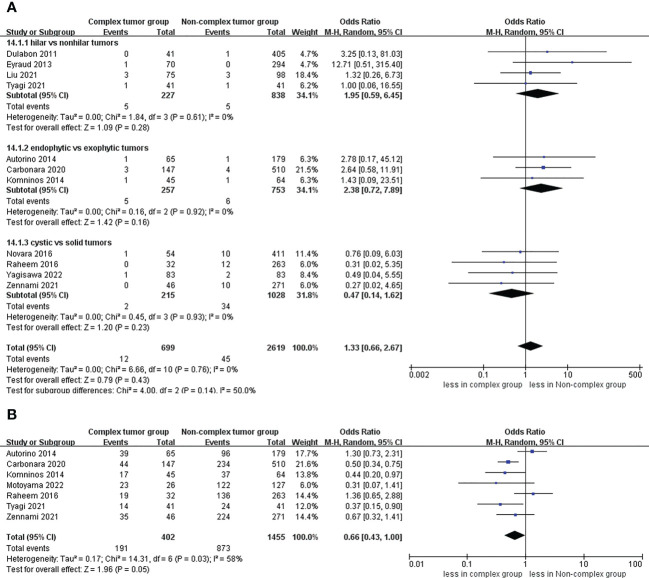
Funnel plot of oncologic outcomes **(A)** recurrence, **(B)** trifecta achievement.

No significant differences were found regarding PSM between the complex tumor and non-complex tumor groups (14 studies; p = 0.19) (17–30). Furthermore, our subgroup analysis also demonstrated that the three subgroups had no statistically significant differences in PSM compared to the non-complex tumor group (p = 0.35; p = 0.18; p = 0.76) (**Figure 8**). Similarly, no statistically significant difference in recurrence was found between the two groups (11 studies; p = 0.43) (**Figure 9**) (17, 18, 20, 21, 23–30), and the subgroup analysis also illustrated no significant difference between the three subgroups and the non-complex group (p = 0.28; p = 0.16; p = 0.23). In terms of trifecta achievement, the pooled results revealed no difference between the two groups (seven studies; p = 0.05) (**Figure 9**) (17, 22–25, 27, 28).

### 3.4 Heterogeneity

Most outcomes showed low to moderate heterogeneity between the included studies. Nevertheless, high heterogeneity was found in warm ischemia time and operative time (I^2 ^= 92%; I^2 ^= 88%).

### 3.5 Sensitivity analysis

Leave-one-out tests were performed to identify changes in heterogeneity in outcomes with high heterogeneity (operative time and warm ischemia time). Finally, no substantial change in heterogeneity was observed among the two perioperative outcomes, indicating that the source of heterogeneity in operative time and warm ischemia time was stable.

### 3.6 Publication bias

A funnel plot was used to assess publication bias, including operative time, warm ischemia time, blood loss, and major complications. The findings showed a roughly tapered distribution of included studies, while there is still some publication bias (**Figure S5**).

## 4 Discussion

This is the first study to assess the perioperative, functional, and oncologic outcomes of RAPN for renal hilar tumors, endophytic renal tumors, and cystic renal tumors. Moreover, some important findings from this analysis need further discussion.

No statistically significant difference in operative time was found between the two groups. Nevertheless, the subgroup analysis reported that renal hilar tumors had a longer operative time than renal nonhilar tumors. The renal hilar tumors had a larger tumor diameter than renal nonhilar tumors, increasing the difficulty of surgery, which might explain the increase in the operative time for renal hilar tumors. In addition to tumor characteristics, many factors influence the operative time, such as the experience of the surgeon and the assistant, BMI, and intraoperative complications (28). Therefore, further research is required to investigate this aspect. In terms of length of hospital stay, no statistical significance was found between the two groups. However, the length of stay for robotic surgery is mostly affected by surgeon expertise and institutional volume rather than surgical methods (31). The combined results demonstrated that the complex tumor group was associated with a longer warm ischemia time than the non-complex tumor group. This finding may be attributed to multiple reasons. First, the increase in warm ischemia time is related to the more challenging dissection, resection and anastomosis of renal hilar tumors and endophytic renal tumors. Second, careful dissection is required for cystic renal tumors to avoid tumor cell spillage, which would increase the warm ischemia time. However, there are certain aspects that warrant our attention, particularly with regard to the optimal duration of warm ischemia during PN, which continues to be a topic of debate within the urological community. Several studies have suggested that warm ischemia time should be limited to 25 or 30 minutes to minimize the risk of renal function impairment (32–34). It is noteworthy that all studies reported an ischemia time of fewer than 30 minutes in the complex tumor group. Considering the above, the ischemia time of the complex tumors is acceptable.

The combined results also revealed that the complex tumor group was associated with more blood loss than the non-complex tumor group, with no statistically significant difference in blood loss among endophytic and cystic renal tumors compared to the non-complex tumor group. In renal hilar tumors, the hilar vessels are responsible for the blood supply to the tumors, supporting their growth (19). Expectedly, these hilar tumors exhibited a larger diameter compared to nonhilar tumors across all the included studies. Furthermore, renal hilar tumors are located close to hilar vessels, which increases the risk of bleeding during the operation. The above reasons might explain the higher blood loss in the hilar tumor group compared to the non-hilar tumor group (19). Although no significant difference was found, larger blood loss was found in endophytic and cystic renal tumors than in the non-complex group in most included studies. The difference might not have been statistically significant due to the small number of included studies in the subgroup analysis. However, the increased blood loss was not likely clinically significant because no significant difference was found in transfusion rates between the two groups. The cumulative analysis revealed no significant difference in the prevalence of conversion to open nephrectomy and radical nephrectomy rates between the two groups. Nevertheless, all the operations in the included studies were performed by experienced surgeons; thus, this outcome should be interpreted with caution.

The meta-analysis revealed that the complex tumor group was associated with more major complications than the non-complex tumor group. The results may be attributed to the complexity of RAPN in tumor resection and reconstruction. However, no patients died due to major complications, suggesting no statistically significant difference in trifecta achievement between the two groups. Moreover, the cumulative analysis revealed no significant difference in intraoperative and overall complications between the two groups. Therefore, higher-level evidence is required to verify our outcomes.

Although the results demonstrated that the complex tumor group was associated with longer warm ischemia time than the non-complex tumor group, no statistically significant difference in eGFR decline was observed between the two groups. It may be due to the following reasons. Recent studies have demonstrated that preoperative renal function and the number of preserved kidneys are the primary factors that are significantly associated with long-term renal function outcomes. In contrast, warm ischemia time has been found to play a relatively minor role in influencing long-term renal function outcomes (35, 36). Furthermore, Fergany et al. (37) showed that age played an important role in the recovery of postoperative long-term renal function. For cystic renal tumors, the extent of renal parenchymal resection may be larger than expected due to the risk of cyst wall damage, which might lead to kidney function loss. Interestingly, the subgroup analysis revealed no significant difference in eGFR decline between cystic renal tumors and solid renal tumors.

The oncologic outcomes are important indicators of surgical quality. Our analysis demonstrated that the complex tumor group had no statistically significant differences in PSM compared to the non-complex tumor group. The PSM rate was 3.10% in the complex tumor group, which is consistent with a high-volume institution that reported PSM rates varying from 0 to 3.7% for RAPN (38). Furthermore, some important aspects of this result need further discussion. First, Marszalek et al. (39) showed that PSM might not be a deciding factor of recurrence. Second, many factors could affect PSM, such as tumor diameter, surgical approach, and tumor stage (40). Therefore, additional studies are required to validate our outcomes. In the included studies, no significant difference in PSM was found between the endophytic tumors and exophytic tumors. In contrast, the endophytic tumors demonstrated slightly higher PSM rates, which might be caused by the higher surgical complexity of endophytic tumors. No statistically significant difference in recurrence was found between the two groups. In cystic renal tumors, cyst rupture might increase the risk of recurrence. Pradere et al. (41) performed a retrospective study evaluating the occurrence of cyst rupture and its effects on recurrence and PSM (50 cyst ruptures out of 268).

Interestingly, there were no recurrence and metastasis in the 50 patients. However, further studies are required to verify this outcome, and particular caution should still be exercised in the manipulation of cystic tumors during the operation. On the other hand, due to insufficient literature, the metastatic recurrence, overall survival, and recurrence-free survival between the two groups cannot be confirmed. Therefore, more studies with a larger sample are required to verify the oncologic results. The difference in trifecta achievement rates between the two groups showed marginal significance (p = 0.05). The trifecta rates were 47.5% for the complex tumor group, which were lower than in cases from the RAPN series for small renal tumors (42). However, many factors could affect the trifecta rates, including tumor diameter and tumor complexity. Our result is consistent with a previous study which reported that trifecta rates of RAPN for highly complex renal tumors (43). On the other hand, the longer warm ischemia time in the complex tumor group seems to be a primary cause affecting trifecta achievement. Nevertheless, trifecta achievement cannot evaluate long-term renal functional and oncologic outcomes, and additional long-term follow-up studies are required to assess the outcomes.

Sagalovich et al. (44) reported the effectiveness and safety between RAPN and open PN for renal hilar tumors. The results of this study demonstrated that RAPN provided similar effectiveness and safety to open PN while it was less invasive. Kara et al. (45) conducted a study to compare the outcomes between RAPN and open PN for completely endophytic renal tumors. The results indicated similar outcomes when performed by experienced surgeons, whereas RAPN exhibited less blood loss and shorter length of stay compared to open PN. Moreover, Pinheiro et al. (46) demonstrated that laparoscopic PN was safe and effective for cystic renal tumors. However, these results remain controversial. Owing to the insufficient literature included, RAPN cannot be compared to other surgical methods. In the included studies, all procedures were performed in large high-volume institutions by experienced laparoscopic and robotic surgeons. Therefore, the outcomes might not be generalized to other institutions, and the results should be interpreted with caution.

However, the limitations of this study should be acknowledged. First, all the studies included in the analysis were non-RCTs, which undoubtedly had potential misclassification bias. Second, no subgroup analysis was performed based on the surgical method (transperitoneal and retroperitoneal), which may lead to subtle differences in outcomes. Third, the tumor complexity (hilar, endophytic, or cystic) might occasionally overlap. Due to insufficient literature, this aspect could not be further analyzed. Last, the follow-up period of some studies is relatively short (3.3-7 months), limiting the comparison between the two groups in terms of renal functional and oncological outcomes.

## 5 Conclusions

The outcomes of the present study demonstrated that RAPN could be a safe and effective procedure for complex tumors (hilar, endophytic, or cystic) with similar perioperative, functional and oncologic outcomes compared to non-complex tumors (nonhilar, exophytic, or solid). Nevertheless, a larger sample size, more long-term follow-up, and data from multicenter studies are required to verify the conclusions.

## Data availability statement

The original contributions presented in the study are included in the article/**Supplementary Material**. Further inquiries can be directed to the corresponding author.

## Author contributions

Protocol development: X-BC, Y-GL and TW; literature search and database creation: X-BC, Y-GL, TW, Z-BD, C-LT and X-DY; formal analysis: X-BC, Y-GL, TW, Z-BD, and X-DY; methodology: X-BC, QZ and X-DY; supervision: X-BC, Y-GL, and X-DY; writing manuscript: X-BC, Y-GL, TW, Z-BD, QZ, C-LT and X-DY. All authors contributed to the article and approved the submitted version.

## Supplementary material

Supplementary Figure 1 Forest plots of perioperative outcome- length of hospital stay.

Supplementary Figure 2 Forest plots of perioperative outcome- transfusion rates.

Supplementary Figure 3 Forest plots of perioperative outcome **(A)** conversion to open nephrectomy rates, **(B)** conversion to radical nephrectomy rates.

Supplementary Figure 4 Forest plots of complication- intraoperative complications.

Supplementary Figure 5 Funnel plot of the studies represented in the meta-analysis **(A)** operative time, **(B)** warm ischemia time, **(C)** blood loss, **(D)** major complications.
