# Supplementary figures and images for: Perioperative, oncologic, and functional outcomes of robot-assisted partial nephrectomy for special types of renal tumors (hilar, endophytic, or cystic): an evidence-based analysis of comparative outcomes
Source: Front Oncol. 2023 Apr 20;13:1178592. doi: 10.3389/fonc.2023.1178592 (PMC10157041; doi:10.3389/fonc.2023.1178592)

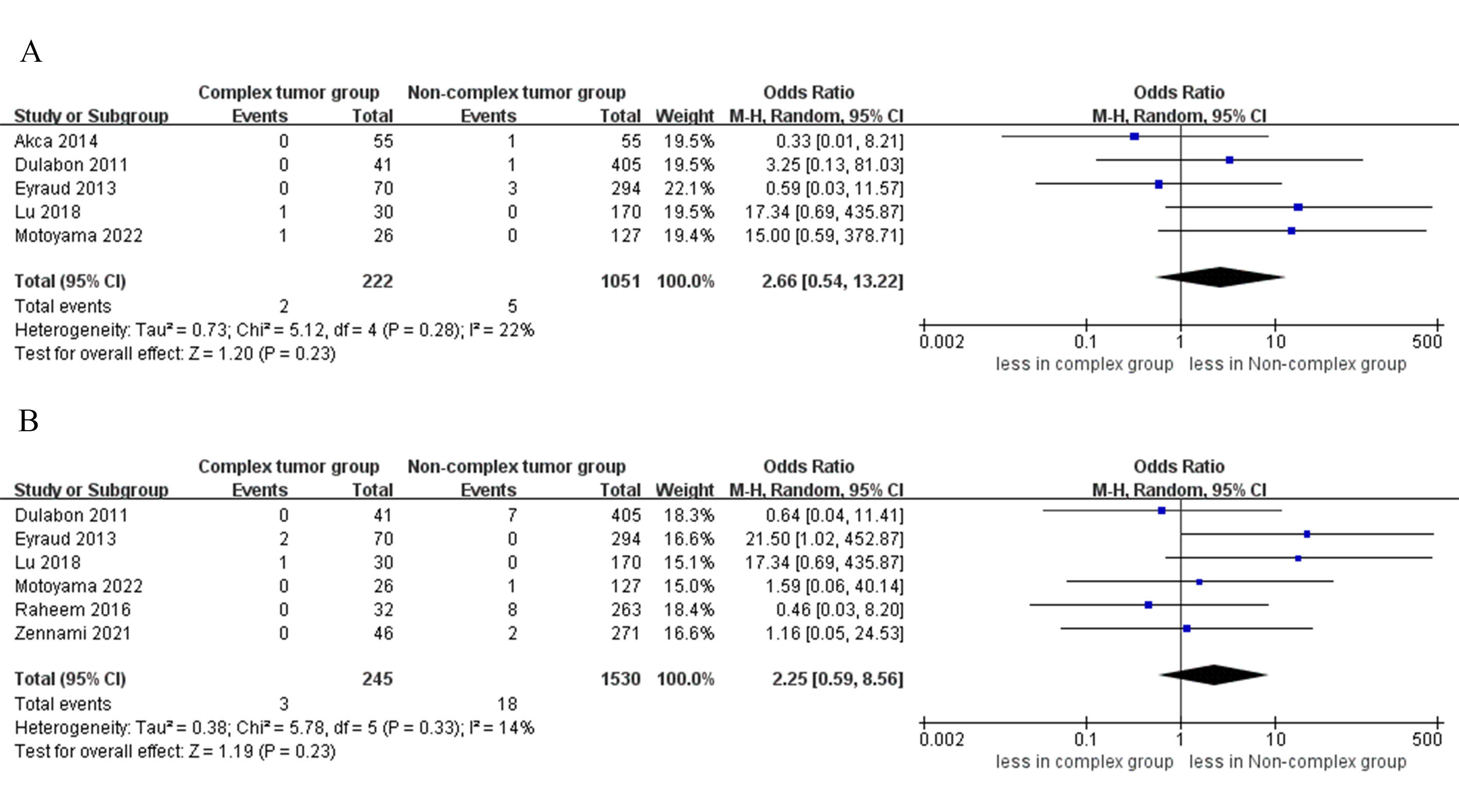

Supplement: Supplementary Figure 3 — Forest plots of perioperative outcome (A) conversion to open nephrectomy rates, (B) conversion to radical nephrectomy rates. [file Image_3.tif]

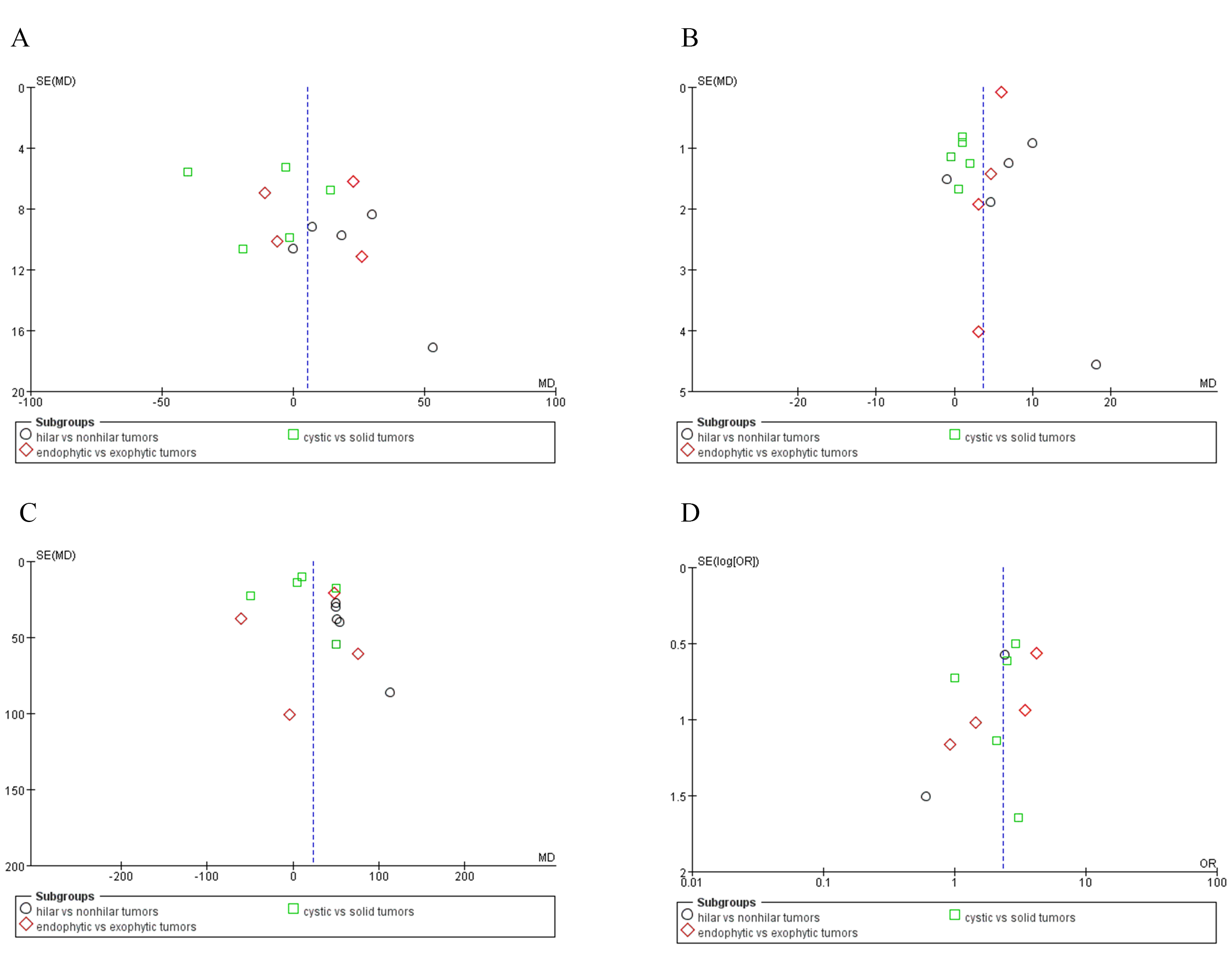

Supplement: Supplementary Figure 5 — Funnel plot of the studies represented in the meta-analysis (A) operative time, (B) warm ischemia time, (C) blood loss, (D) major complications. [file Image_5.tif]
