# Supplementary material for: Perioperative, oncologic, and functional outcomes of robot-assisted partial nephrectomy for special types of renal tumors (hilar, endophytic, or cystic): an evidence-based analysis of comparative outcomes
Source: Front Oncol. 2023 Apr 20;13:1178592. doi: 10.3389/fonc.2023.1178592 (PMC10157041; doi:10.3389/fonc.2023.1178592)
Supplement: Supplementary file 6 [file Table_1.docx]

| **Table S1 Comparison of baseline patient** | | | |  |
| --- | --- | --- | --- | --- |
| Baseline characteristic | Complex VS Non-complex tumor group | Heterogeneity I^2^ (%) | *p* value |  |
| Age WMD (95% CI) | -1.76(-3.57 to 0.05) | 79 | 0.06 |  |
| BMI WMD (95% CI) | -0.35(-0.80 to 0.11) | 29 | 0.14 |  |
| Tumor diameter (95% CI) | 0.25(-0.19 to 0.68) | 89 | 0.26 |  |
| Preoperative eGFR WMD (95% CI) | 0.25(-2.56 to 3.07) | 62 | 0.86 |  |
| eGFR: estimated glomerular filtration rate | | | |  |
|  |  |  |  |  |
|  |  |  |  |  |
