# Supplementary material for: Perioperative, oncologic, and functional outcomes of robot-assisted partial nephrectomy for special types of renal tumors (hilar, endophytic, or cystic): an evidence-based analysis of comparative outcomes
Source: Front Oncol. 2023 Apr 20;13:1178592. doi: 10.3389/fonc.2023.1178592 (PMC10157041; doi:10.3389/fonc.2023.1178592)
Supplement: Supplementary file 7 [file Table_2.docx]

| **Table S2 The risk of bias (Non-RCTs)-ROBINS-I** | | | | | | | | | | | | | | |  |
| --- | --- | --- | --- | --- | --- | --- | --- | --- | --- | --- | --- | --- | --- | --- | --- |
| Bias domain | Tyagi | Liu | Lu | Eyraud | Dulabon | Motoyama | Carbonara | Komninos | Autorino | Yagisawa | Zennami | Raheem | Novara | Akca |  |
|  |  |  |  |  |  |  |  |  |  |  |  |  |  |  |  |
| Bias due to confounding | Moderate | Moderate | Moderate | Moderate | Moderate | Moderate | Moderate | Moderate | Moderate | Moderate | Moderate | Moderate | Moderate | Moderate |  |
|  |  |  |  |  |  |  |  |  |  |  |  |  |  |  |  |
| Bias in selection of participants into the study | Low | Low | Low | Low | Low | Low | Low | Low | Moderate | Low | Low | Moderate | Low | Low |  |
|  |  |  |  |  |  |  |  |  |  |  |  |  |  |  |  |
| Bias in classification of interventions | Low | Low | Moderate | Low | Low | Moderate | Low | Low | Low | Low | Low | Low | Low | Low |  |
|  |  |  |  |  |  |  |  |  |  |  |  |  |  |  |  |
| Bias due to deviations from intended interventions | Moderate | Moderate | Low | Moderate | Low | Moderate | Moderate | Low | Moderate | Low | Low | Low | Moderate | Moderate |  |
|  |  |  |  |  |  |  |  |  |  |  |  |  |  |  |  |
| Bias due to missing data | Moderate | Low | Moderate | Low | Moderate | Low | Low | Moderate | Low | Low | Moderate | Moderate | Low | Low |  |
|  |  |  |  |  |  |  |  |  |  |  |  |  |  |  |  |
| Bias in measurement of outcomes | Low | Moderate | Low | Moderate | Low | Moderate | Low | Moderate | Low | Moderate | Low | Low | Moderate | Moderate |  |
|  |  |  |  |  |  |  |  |  |  |  |  |  |  |  |  |
| Bias in selection of the reported result | Moderate | Moderate | Moderate | Moderate | Moderate | Moderate | Moderate | Moderate | Moderate | Moderate | Moderate | Moderate | Moderate | Moderate |  |
|  |  |  |  |  |  |  |  |  |  |  |  |  |  |  |  |
| Overall bias | Moderate | Moderate | Moderate | Moderate | Moderate | Moderate | Moderate | Moderate | Moderate | Moderate | Moderate | Moderate | Moderate | Moderate |  |
|  |  |  |  |  |  |  |  |  |  |  |  |  |  |  |  |
